# Whole-Genome Sequencing Elucidates the Superior Fermentation Performance, Flavor Formation, and Nutritional Remodeling Mechanisms of *Lactococcus garvieae* DZ for Fermented Butter Production

**DOI:** 10.3390/microorganisms14102163

**Published:** 2026-09-25

**Authors:** Dan-Dan Liu, Jia-Wei Xu, Jia-Yu Ma, Xi Yu, Lei Qian, Ye-Ni Zhang

**Affiliations:** 1 College of Food Science and Bioengineering, Tianjin Agricultural University, Tianjin 300392, China; liudandan20001013@163.com (D.-D.L.); xu_jiawei1023@163.com (J.-W.X.);; 2 Shandong Dezheng Dairy Co., Ltd., Weihai 264400, China; 3 College of Agronomy and Resources and Environment, Tianjin Agricultural University, Tianjin 300392, China

**Keywords:** *Lactococcus garvieae*, whole-genome sequencing, fermented butter, flavor formation, fatty acid metabolism, probiotic properties

## Abstract

*Lactococcus garvieae* DZ is a specialized fermentation strain isolated from raw water buffalo milk, previously shown to exhibit excellent fermentation performance and significantly enhance the flavor and nutritional quality of butter. To elucidate the molecular basis of these properties, this study employed a combination of second- and third-generation sequencing technologies to complete whole-genome sequencing and functional annotation. The genome consists of one circular chromosome (2.15 Mb, GC content 38.81%) and three plasmids, encoding a total of 2123 genes. Through annotation against KEGG, CAZy, and other databases, this study systematically identified flavor-forming pathways including lactose degradation, glycolysis, diacetyl synthesis, and short- and medium-chain fatty acid accumulation, providing a molecular explanation for the explosive accumulation of characteristic flavor compounds (2,3-butanedione reached 6188 μg·kg^−1^). The presence of oleate hydratase is consistent with the decrease in oleic acid proportion. Lipolytic enzymes and the antioxidant system may facilitate the release and oxidative protection of unsaturated fatty acids, thereby contributing to the overall increase in PUFAs and MUFAs such as CLA. Genes identified in the genome—including those encoding Na^+^/H^+^ antiporters, bile salt hydrolase, and molecular chaperone systems—are consistent with the strain’s excellent gastrointestinal tolerance (94.75% survival at pH 3.0 and 40.43% survival in 0.3% bile salts); the annotation of the *β*-galactosidase gene also corresponded to its measured enzymatic activity (0.271 U·mL^−1^). From a safety perspective, no transferable antibiotic resistance genes or typical virulence factors were identified in the genome. Although a hemolysin III gene was annotated, its encoded product is annotated only as a predicted membrane protein and lacks a known toxin catalytic domain; blood agar plate assays confirmed that strain DZ exhibits *γ*-hemolysis (non-hemolytic), with phenotype consistent with genotype, ruling out the risk of hemolytic virulence. This study elucidates, at the genomic level, the genetic basis underlying the superior fermentation performance, probiotic properties, and safety of strain DZ, providing a theoretical foundation for its industrial application.

## 1. Introduction

Fermented butter, also known as cultured butter, is a dairy fat product produced through the fermentation of cream by microorganisms such as lactic acid bacteria [1]. Compared to conventional butter, fermented butter exhibits a richer and more complex flavor profile due to the metabolic activities of lactic acid bacteria, and offers potential health benefits [2,3]. Its typical production process involves inoculating cream with lactic acid bacteria for fermentation, followed by ripening and centrifugation; flavor development primarily occurs during the cream fermentation stage.

Lactic acid bacteria are the core determinant of fermented butter quality; the metabolic characteristics of different species and strains directly influence the product’s flavor profile, fatty acid composition, and functional properties [4,5,6,7]. Currently, the starter cultures commonly used in fermented butter production mainly include *Lactococcus lactis*, *Leuconostoc mesenteroides*, and members of the genus *Lactobacillus*, with strain selection having long focused on traditional traits such as acid production rate and aroma-forming capacity [8]. However, as consumer demand for natural and nutritionally functional foods continues to grow, the screening criteria for starter strains have expanded beyond mere flavor contribution to encompass active shaping of the product’s nutritional quality—for example, the enrichment of polyunsaturated fatty acids and the generation of beneficial lipid metabolites [9,10]. This shift places greater demands on the systematic exploration of strain genetic resources and the precise characterization of functional genes.

Whole-genome sequencing technology provides a powerful tool for elucidating the genetic basis of desirable microbial phenotypes. By obtaining the complete genome sequence of a strain and performing functional annotation, researchers can systematically identify key functional genes associated with specific traits, thereby establishing “genotype–phenotype” associations [11,12]. In recent years, whole-genome sequencing has been widely applied in functional gene mining, safety assessment, and metabolic pathway reconstruction of lactic acid bacteria, laying a molecular foundation for the targeted breeding and metabolic engineering of starter strains [13,14].

*Lactococcus garvieae* is a species with a dual ecological and clinical profile. It is a well-recognized fish pathogen causing lactococcosis in aquaculture, and it has also been reported as an opportunistic human pathogen, particularly in immunocompromised individuals. At the same time, *L. garvieae* is widely distributed in traditional fermented foods. Wang et al. [15] isolated *L. garvieae* TC-6 from traditional air-dried meat and used it as a starter culture for beef fermentation, where it improved protein degradation and flavor quality. Zhao et al. [16] performed comparative genomic analysis of *L. garvieae* from Baijiu pit mud and reported that these strains were safe at the genomic level based on VFDB annotation, while also noting the possibility of environment-induced toxin gene expression and the occurrence of horizontal gene transfer. These studies indicate that *L. garvieae* is not only a fish pathogen and an opportunistic human pathogen, but also a functionally important microorganism in traditional fermented foods. Therefore, the safety of strain DZ should be assessed at the strain level and interpreted with caution. It should be noted that *Lactococcus garvieae* has not been granted QPS (Qualified Presumption of Safety) status by the European Food Safety Authority (EFSA). Therefore, unlike some well-established dairy lactic acid bacteria such as *Lactococcus lactis*, *L. garvieae* cannot be regarded as safe by default for food or industrial applications, and its safety must be assessed at the strain level. Our laboratory previously isolated and characterized a strain of *Lactococcus garvieae* designated DZ (CGMCC No. 37371) from raw water buffalo milk. This strain exhibits outstanding fermentation performance in fermented butter production: under optimized process conditions, substantial accumulation of characteristic flavor compounds occurs in the fermented butter—specifically, 2,3-butanedione reached 6188.24 μg·kg^−1^, 2,3-pentanedione reached 4660.37 μg·kg^−1^, and acetoin reached 2389.94 μg·kg^−1^—while the levels of various esters and lactones were also markedly increased. Additionally, strain DZ possesses excellent probiotic-related properties, including strong gastrointestinal tolerance (94.75% survival at pH 3.0 and 40.43% survival in 0.3% bile salts), *β*-galactosidase activity (0.271 U·mL^−1^), and no hemolytic activity. Fatty acid analysis further revealed that fermentation with strain DZ significantly remodels the fatty acid profile of butter: conjugated linoleic acid increased by 40% (to 0.77%), *α*-linolenic acid increased by 21% (to 1.03%), while the proportion of palmitic acid decreased significantly. These phenotypic characteristics demonstrate that strain DZ is a specialized strain for fermented butter production with dual potential for flavor enhancement and nutritional remodeling.

However, the molecular mechanisms underlying the superior phenotypes of strain DZ remain unclear: What is the genetic basis for its high production of diacetyl and short- and medium-chain fatty acids? Which key genes are involved in the nutritional remodeling of the butter fatty acid profile? Which genes mediate its excellent gastrointestinal tolerance? Are there transferable antibiotic resistance genes or virulence factors in its genome? Addressing these scientific questions will not only help understand the superior traits of strain DZ at the molecular level but also provide a theoretical foundation for subsequent targeted improvement and industrial application of this strain.

Against this background, this study employed a combined second-generation (Illumina) and third-generation (PacBio) whole-genome sequencing strategy for genome assembly, annotation, and functional gene mining of strain DZ. Through systematic comparisons against the NR, KEGG, COG, GO, CAZy, CARD, and VFDB databases, this study aimed to: (1) characterize the general genomic features of strain DZ; (2) identify key genes associated with flavor formation (diacetyl/acetoin pathway, short- and medium-chain fatty acid synthesis, and ester production); (3) identify key genes related to nutritional remodeling (conjugated linoleic acid accumulation, increase in polyunsaturated fatty acids, and regulation of health-risk fatty acids); (4) identify key genes associated with probiotic properties (acid resistance, bile salt resistance, intestinal colonization, and antimicrobial activity); and (5) systematically assess the biosafety of strain DZ (antibiotic resistance genes, virulence factors, and hemolytic activity). This study reveals, at the genomic level, the molecular basis of the superior fermentation, flavor formation, nutritional remodeling, and probiotic characteristics of strain DZ, providing a theoretical foundation for its industrial application and subsequent targeted improvement, as well as a scientific reference for mining genetic resources of superior strains for fermented butter production.

## 2. Materials and Methods

### 2.1. Strain

*Lactococcus garvieae* DZ (CGMCC No. 37371) is a strain isolated, identified, and preserved by our laboratory from raw water buffalo milk.

### 2.2. Main Materials and Reagents

MRS broth and MRS agar media (Beijing Aoboxing Biotechnology Co., Ltd., Beijing, China); bacterial genomic DNA extraction kit (Beijing Solarbio Technology Co., Ltd., Beijing, China); agarose (Shanghai Macklin Biochemical Technology Co., Ltd., Shanghai, China); DNA Marker (Takara Biomedical Technology (Beijing) Co., Ltd., Beijing, China). All other reagents were of domestic analytical grade.

### 2.3. Main Instruments and Equipment

Qubit 3.0 Fluorometer (Invitrogen, Carlsbad, CA, USA), Agilent 2100 Bioanalyzer (Agilent Technologies, Santa Clara, CA, USA), Covaris g-TUBE fragmentation device (Covaris, Inc., Woburn, MA, USA), PacBio Sequel IIe sequencing platform (Pacific Biosciences, Menlo Park, CA, USA), and Illumina NovaSeq sequencing platform (Illumina, San Diego, CA, USA). All other equipment consisted of routine laboratory instruments.

### 2.4. Strain Cultivation and Genomic DNA Extraction

Strain DZ was activated and cultured for two generations in MRS medium at 37 °C; the resulting secondary-activated bacterial pellet was collected for genomic DNA extraction. Genomic DNA was extracted using a bacterial genomic DNA extraction kit according to the manufacturer’s instructions. DNA integrity was verified by 1% agarose gel electrophoresis, and the extracted genomic DNA was stored at −20 °C for subsequent use.

### 2.5. Sequencing Library Construction and Sequencing

#### 2.5.1. Second-Generation Sequencing (Illumina) Library Construction and Sequencing

A total of 100 ng of genomic DNA was randomly fragmented into fragments smaller than 500 bp using a Covaris S220 ultrasonic disruptor(Covaris, Inc., Woburn, MA, USA). End repair (including 5′-end phosphorylation and 3′-end A-tailing) was performed using the End Prep Enzyme Mix, followed by adapter ligation to both ends. Target fragments were purified using magnetic beads and amplified using P5 and P7 primers. Library quality was assessed using an Agilent 2100 Bioanalyzer (Agilent Technologies, Palo Alto, CA, USA), and library concentration was determined using a Qubit 3.0 fluorometer (Invitrogen, Waltham, MA, USA). Validated libraries were used for cluster generation and sequencing.

#### 2.5.2. Third-Generation Sequencing (PacBio) Library Construction and Sequencing

A total of 5–10 μg of high-quality genomic DNA was randomly fragmented into approximately 10 kb fragments using a Covaris g-TUBE device (Covaris, Inc., Woburn, MA, USA), followed by SMRTbell library construction using the SMRTbell Prep Kit 3.0 (Pacific Biosciences, Menlo Park, CA, USA). The prepared SMRTbell library, along with sequencing primers and polymerase, was loaded onto the sequencing chip of the PacBio Sequel IIe platform via diffusion for sequencing. The PacBio reads used for assembly were long subreads generated from the SMRTbell library.

### 2.6. Genome Assembly and Correction

Genome assembly was performed using third-generation sequencing data generated on the PacBio Sequel IIe platform, with preliminary assembly conducted using Hifiasm (version 0.13-r308) and Canu (version 1.7) software. The sequencing coverage was approximately 20× for PacBio and approximately 1005× for Illumina. Raw sequencing reads were quality-controlled using second-generation sequencing data generated on the Illumina NovaSeq platform, with adapter sequences and low-quality reads removed. The quality-controlled second-generation sequencing data were then aligned to the preliminary assembly results, and the assembled genome was further refined using Pilon software (version 1.24) to obtain the final assembly. The preliminary assembly contained multiple contigs, and after polishing with Pilon, the final assembly was resolved into four circularized replicons: one chromosome and three plasmids. The final assembly was evaluated for completeness and contamination, and all four replicons (one chromosome and three plasmids) were confirmed to be circular closed.

### 2.7. Genome Annotation and Protein Feature Prediction

#### 2.7.1. Coding Gene Prediction

Coding genes in the assembled genomic sequences were predicted using Prodigal (version 2.6.3) software.

#### 2.7.2. Plasmid Genome Annotation

Functional elements in plasmid genomes—including promoters, terminators, and resistance genes—were annotated using pLannotate software (version 1.2.0).

#### 2.7.3. Non-Coding RNA Prediction

tRNA and rRNA regions were predicted using tRNAscan-SE (version 2.0.12) and Barrnap (version 0.9) software, respectively; other non-coding RNAs were identified through sequence alignment against the Rfam (version 12.0) database.

#### 2.7.4. Gene Functional Annotation

Based on the predicted protein sequences of the encoded genes, Diamond (version 0.8.15) and BLAST (version 2.3.0) software were used to perform sequence alignment against the NR, KEGG, COG, GO, CAZy, Swiss-Prot, Pfam, CARD, and VFDB databases (E-value < 1 × 10^−5^); the best-matching results were selected for gene functional annotation.

#### 2.7.5. Secretory Protein Prediction

Signal peptides were predicted using the SignalP (version 4.1) online tool, and transmembrane domains were predicted using the TMHMM (version 2.0) online tool. Proteins containing signal peptides but no transmembrane domains were classified as secreted proteins.

### 2.8. Repeat Sequence Analysis

RepeatModeler (version 1.0.8) was used for de novo prediction of genomic repetitive sequences. The analysis was conducted in two steps: first, RECON (version 1.08) and RepeatScout (version 1.0.5) were employed to identify potential repetitive sequences; the results were then optimized and a library was constructed using RepeatModeler. Second, RepeatMasker (version 4.0.6) was used to perform search and analysis of repetitive sequences within the target genome. Additionally, NUCmer (version 3.1) was employed to detect repetitive sequences within the genome and to screen for long fragment repeats with a length ≥ 100 bp for subsequent circular mapping.

### 2.9. Genome Circular Diagram Construction

Genomic circular diagrams depicting genes, ncRNA, GC content, and repetitive sequence information were generated using Circos (version 0.69-3) software.

### 2.10. Data Processing

Raw sequencing data underwent base calling and quality control using Bcl2fastq (v2.20) to generate raw sequencing data in FASTQ format. Quality control was performed on the raw data using fastp (v0.23.0) to remove adapter sequences and low-quality reads. Statistical tables containing the genome assembly and annotation results were compiled using Microsoft Excel 2019.

### 2.11. Phenotypic Assays

The phenotypic data for acid tolerance, bile salt tolerance, *β*-galactosidase activity, antimicrobial activity, hemolysis, and antibiotic susceptibility were obtained from our previous study [17]. The experimental protocols, culture conditions, replicates, controls, and statistical methods are described in detail in that publication.

Briefly, acid tolerance was assessed by inoculating strain DZ at 3% (*v/v*) into MRS broth at pH 3.0 and incubating at 37 °C for 3 h, followed by viable counting on MRS agar; the survival rate was calculated by comparing the viable counts before and after acid treatment. Bile salt tolerance was assessed in MRS broth containing 0.3% (*m/v*) bovine bile salt at 37 °C for 4 h, and the survival rate was calculated by comparing the viable counts before and after bile salt treatment. *β*-Galactosidase activity was determined by the ONPG method at 420 nm, and one unit (U) was defined as the amount of enzyme releasing 1 μmol of o-nitrophenol per minute. Antimicrobial activity was determined by the Oxford cup method against seven foodborne pathogens, with sterile MRS broth as the negative control. Hemolytic activity was assessed on Columbia blood agar plates containing defibrinated sheep blood, with *Pseudomonas aeruginosa* as the positive control and an uninoculated Columbia blood agar plate as the negative control. Antibiotic susceptibility was determined by the disk diffusion method. This method was chosen because it is simple, cost-effective, and reproducible, and it is widely used for antimicrobial susceptibility testing of lactic acid bacteria. The disk potencies were as follows: ampicillin (10 μg), tetracycline (30 μg), chloramphenicol (30 μg), erythromycin (15 μg), clindamycin (2 μg), gentamicin (10 μg), streptomycin (10 μg), ciprofloxacin (5 μg), vancomycin (30 μg), and rifampicin (5 μg). The results were interpreted according to CLSI M100-Ed35 (2025).

All experiments were performed in triplicate, and data are presented as mean ± SD. Statistical analysis was performed using SPSS 25.0 with one-way ANOVA followed by Tukey’s HSD test (*p* < 0.05).

### 2.12. Flavor and Fatty Acid Analysis

The volatile flavor compounds and fatty acid composition were obtained from our previous study [18]. Briefly, volatile flavor compounds were determined by HS-SPME-GC-MS, and fatty acid composition was determined by BF_3_ methylation followed by GC-MS. All experiments were performed in triplicate, and data are presented as mean ± SD. Statistical analysis was performed using SPSS 25.0 with one-way ANOVA followed by Tukey’s HSD test (*p* < 0.05).

## 3. Results and Discussion

### 3.1. General Genomic Characteristics

Whole-genome sequencing of strain DZ was performed using a combined approach involving Illumina NovaSeq and PacBio Sequel IIe platforms [19]. After filtering, 15,437,008 high-quality second-generation reads (2.32 Gb, Q30 = 92.43%) and 14,400 third-generation PacBio subreads (45.59 Mb, N50 = 3479 bp) were obtained. The N50 of 3479 bp refers to the raw PacBio subreads, not the final assembly. After quality filtering, 15,347,962 clean reads (2.29 Gb) were retained, with Q20 = 97.47% and Q30 = 92.64%, corresponding to a read retention rate of 99.42% and a base retention rate of 99.00%. The N content was only 7.43–7.90 ppm.

After assembly and refinement, the DZ genome consists of one circular chromosome (2,146,103 bp) and three circular plasmids (pDZ1, 44,601 bp; pDZ2, 15,330 bp; pDZ3, 74,115 bp). All four replicons were assembled as circular closed sequences, indicating that the assembly is complete and that no gaps remain. Mapping statistics showed that 14,396 of 14,400 reads (99.97%) were mapped to the genome, with 100% genome coverage and a mean depth of 19.97×. No contamination or additional contigs were detected. The chromosome measures 2,146,103 bp in length with a GC content of 38.81%; the three plasmids are designated as pDZ1 (44,601 bp, GC content 36.04%), pDZ2 (15,330 bp, GC content 34.26%), and pDZ3 (74,115 bp, GC content 34.40%). Functional annotation of the three plasmids revealed that pDZ1 mainly carries a lactose metabolism gene cluster (*lacABCGXFDR*), along with transposase genes (*tnp1*), a resolvase gene (*resR*), and a mercury resistance regulator gene (*merR1*); pDZ2 mainly carries transposase genes (*tnp1*), a resolvase gene (*tnpR*), and a ketopantoate reductase gene (*PANE_LACLA*); and pDZ3 mainly carries a lactococcin secretion system (*lcnC/lcnD*), a DNA methylase gene (*hsdFM*), and transposase genes (*tnp1*). No acquired antibiotic resistance genes or typical virulence factor genes were identified on the plasmids. The genomic circle map (Figure 1) provides a visual overview of the distribution of genes, ncRNAs, and GC content along the chromosome [20]. The genome encodes a total of 2123 genes, with the total coding region length amounting to 1,884,552 bp, representing 82.6% of the total genome length; the average length of the encoded genes is 887.68 bp, and the N50 value is 1146 bp. Repeat sequence analysis revealed that no typical transposable elements or simple repetitive sequences were detected in the DZ genome; only a small number of long fragment repeats (≥100 bp) were present on the chromosome, suggesting high genetic stability of this strain [21,22].

The COG functional classification results (Figure 2) indicate that genes in the DZ genome are primarily enriched in categories such as carbohydrate metabolism and transport (G), amino acid metabolism and transport (E), translation and ribosomal structure (J), and cell wall/membrane biogenesis (M), which lay the foundation for its efficient fermentation performance.

### 3.2. Genes Associated with Carbohydrate Metabolism and Flavor Formation

To elucidate the molecular basis of the high-yield characteristic flavor compounds in strain DZ, this study conducted a systematic analysis of carbohydrate metabolism-related genes in its genome. The KEGG metabolic pathway annotation results (Figure 3) reveal that genes in the DZ genome are predominantly enriched in pathways such as carbohydrate metabolism, amino acid metabolism, and membrane transport. The CAZy classification of carbohydrate-active enzymes (Figure 4) indicates that glycoside hydrolases (GH) and glycosyltransferases (GT) represent the two most abundant enzyme classes, providing an enzymatic foundation for lactose degradation and flavor compound synthesis [23]. Data in Section 3.2, Section 3.3, Section 3.4 and Section 3.5 are presented as mean ± SD (*n* = 3) and were obtained from our previous studies [17,18]. Statistical significance was set at *p* < 0.05.

#### 3.2.1. Lactose Degradation and Glycolytic Pathway

The DZ genome fully encodes the lactose degradation and galactose metabolism pathways. The *β*-galactosidase gene (*bgaB*, 1_1472) hydrolyzes lactose into glucose and galactose. Galactose then enters the Leloir pathway, where it is sequentially converted into glucose-1-phosphate via the catalysis of galactokinase (*galK*, 1_1778), galactose-1-phosphate uridylyltransferase (*galT*, 1_1352, 1_1777), UDP-glucose-4-epimerase (*galE*, 1_119, 1_1776, 1_1868), and UDP-glucose pyrophosphorylase (*galU*, 1_677) [24]. The glucose released from lactose hydrolysis and the glucose-1-phosphate generated via the Leloir pathway are then converted separately: glucose is phosphorylated to glucose-6-phosphate by glucokinase (*glk,* 1_609, 1_1814, 1_830), while glucose-1-phosphate is isomerized to glucose-6-phosphate by phosphoglucomutase (*pgm*, 1_1656) [25]. These two metabolites then converge and enter the glycolytic pathway together.

All genes associated with the glycolytic pathway were identified in the DZ genome, including 6-phosphofructokinase (*pfkA*, 1_693), fructose-bisphosphate aldolase (*fba*, 1_1505, 1_1735), glyceraldehyde-3-phosphate dehydrogenase (*gapA*, 1_2101), phosphoglycerate kinase (*pgk*, 1_1882), phosphoglycerate mutase (*gpmA*, 1_199, 1_504), enolase (*eno*, 1_1544), and pyruvate kinase (*pyk*, 1_694). The integrity of these genes indicates that strain DZ possesses a complete glycolytic capacity to convert lactose into pyruvate [26].

#### 3.2.2. Diacetyl/Acetoin Metabolic Pathway

Pyruvate serves as a critical node in flavor compound synthesis. A complete diacetyl/acetoin metabolic pathway was identified in the DZ genome: the acetolactate synthase gene (*alsS*, 1_1078) catalyzes the condensation of two pyruvate molecules to form *α*-acetolactate; under aerobic conditions, *α*-acetolactate can be converted into diacetyl via non-enzymatic oxidative decarboxylation [27]; alternatively, *α*-acetolactate can also be decarboxylated to produce acetoin under the action of the acetolactate decarboxylase gene (*alsD*, 1_853, 1_1039). Additionally, the butanediol dehydrogenase gene (*bdhAB*, 1_1089, 1_1316, 1_1872, 1_1875, 1_951) catalyzes the two-step redox reaction of acetoin to generate 2,3-butanediol, thereby regulating the accumulation level of acetoin [28]. Thus, *α*-acetolactate serves as a common precursor for both diacetyl and acetoin synthesis; its metabolic flux directly influences the accumulation ratio of these two flavor compounds.

The integrity of this pathway provides a molecular-level explanation for the substantial accumulation of characteristic flavor compounds in DZ-fermented butter: the 2,3-butanedione content reached 6188.24 ± 278.52 μg·kg^−1^, which was higher than that in commercial butter (943.81 ± 45.91 μg·kg^−1^); the acetoin content was 2389.94 ± 24.86 μg·kg^−1^, whereas none was detected in commercial products. Additionally, high levels of 2,3-pentanedione (4660.37 ± 417.66 μg·kg^−1^) were detected in DZ-fermented butter but not in commercial products; its synthesis may be related to the substrate promiscuity of acetolactate synthase.

#### 3.2.3. Short- and Medium-Chain Fatty Acid Synthesis System

Short- and medium-chain fatty acids (C4–C10) are important contributors to the buttery and cheese-like flavor profile. The DZ genome encodes the core enzyme system involved in fatty acid synthesis, including acetyl-CoA carboxylase (*accA* 1_554, *accB* 1_549, *accC* 1_551, *accD* 1_553), *β*-ketoacyl-ACP synthase III (*fabH* 1_544), ACP-S-malonyltransferase (*fabD* 1_546), 3-oxoacyl-ACP reductase (*fabG* 1_1313, 1_547, 1_561), *β*-ketoacyl-ACP synthase II (*fabF*1_548), 3-hydroxyacyl-ACP dehydratase (*fabZ* 1_550, 1_1663), enoyl-ACP reductase I (*fabI* 1_1661), and medium-chain acyl-ACP thioesterase (*tesA* 1_319, 1_947) [29]. This thioesterase serves as a key enzyme that regulates C8–C10 medium-chain fatty acid synthesis, terminates carbon chain elongation, and releases free fatty acids, providing a crucial enzymatic foundation for the accumulation of medium-chain fatty acids in strain DZ [30].

In line with this, metabolite profiling results revealed that, compared to non-fermented butter, the content of C4–C10 fatty acids in DZ-fermented butter was significantly increased: butyric acid (C4:0) rose from 1603.18 ± 108.04 μg·kg^−1^ to 3673.07 ± 102.49 μg·kg^−1^ (2.3-fold), hexanoic acid (C6:0) increased from 2079.27 ± 417.74 μg·kg^−1^ to 7418.06 ± 1050.72 μg·kg^−1^ (3.6-fold), octanoic acid (C8:0) increased from 1971.89 ± 156.90 μg·kg^−1^ to 7062.42 ± 1396.43 μg·kg^−1^ (3.6-fold), and decanoic acid (C10:0) increased from 1917.20 ± 64.61 μg·kg^−1^ to 5570.59 ± 1127.09 μg·kg^−1^ (2.9-fold).

#### 3.2.4. Ester Synthesis-Related Enzyme Systems

Ester compounds impart a complex flavor balance featuring fruity, cheesy, and creamy notes to the butter. Multiple key precursor supply enzyme systems involved in ester synthesis were identified in the DZ genome.

Metabolite profiling results revealed that DZ-fermented butter contained elevated levels of ethyl lactate, ethyl acetate, and various ethyl fatty acid esters; specifically, the ethyl lactate concentration reached 358.44 ± 21.75 μg·kg^−1^, while the ethyl acetate concentration reached as high as 6736.62 ± 923.83 μg·kg^−1^—both compounds were detected exclusively in DZ-fermented butter and were not detected in either commercial butter or non-fermented butter; the acetate content was 4354.32 ± 353.47 μg·kg^−1^, which is 5.9 times higher than that in non-fermented butter; furthermore, the concentrations of ethyl butyrate (211.21 ± 11.19 μg·kg^−1^), ethyl octanoate (430.16 ± 63.55 μg·kg^−1^), and ethyl decanoate (189.52 ± 12.30 μg·kg^−1^) were also higher than those in non-fermented butter.

The accumulation profile of these ester products is highly consistent with the integrity of the ester synthesis precursor supply enzyme system within the DZ genome. The lactate dehydrogenase gene (*ldh*, 1_161, 1_695) catalyzes the reduction of pyruvate to lactate, thereby providing an acidic precursor for ethyl lactate synthesis; the pyruvate oxidase gene (*spxB*, 1_1006) catalyzes the oxidation of pyruvate to acetate, supplying a precursor for ethyl acetate synthesis; the acetyl-CoA synthetase gene (*acs*, 1_1368) activates acetate into acetyl-CoA; and the bifunctional acetaldehyde/alcohol dehydrogenase gene (*adhE*, 1_1918) sequentially catalyzes the conversion of acetyl-CoA to acetaldehyde, followed by its reduction to ethanol, thereby providing alcohol precursors for the synthesis of various ethyl esters [31]. Short- and medium-chain fatty acids—such as butyric acid, octanoic acid, and decanoic acid—may be synthesized and supplied by the fatty acid synthase system (FAS).

Notably, no typical alcohol acyltransferase (AAT) gene was identified in the DZ genome, suggesting that ester synthesis in this strain primarily relies on non-canonical metabolic pathways. However, the genome contains multiple annotated *α*/*β* hydrolases (aes 1_640, 1_815, *pldB* 1_203) and esterases (*estA*1_436); these proteins can catalyze the reverse reaction of hydrolysis (esterification) in the fermentation environment and may also participate in ester synthesis through their broad acyltransferase activity [32]. The abundant ester products obtained from DZ-fermented butter not only reflect the unique metabolic potential of this strain but also provide an important foundation for further exploration of novel ester synthases and the elucidation of non-canonical ester synthesis pathways.

#### 3.2.5. Lactone Compound Synthesis

Lactones impart a rich coconut, creamy, and peachy aroma to the butter. Metabolite profiling results revealed a significant increase in lactone content in DZ-fermented butter: the *γ*-hexalactone content reached 5602.67 ± 636.57 μg·kg^−1^ (1.7 times that of commercial products), while the *δ*-octalactone content reached 2109.58 ± 67.52 μg·kg^−1^ (4.6 times that of non-fermented butter).

Lactones are typically synthesized through intramolecular esterification of hydroxy fatty acids [33]. The DZ genome harbors an oleate hydratase gene (*ohyA*, 1_1392), which converts unsaturated fatty acids such as oleic acid into hydroxy fatty acids, thereby providing precursors for lactone synthesis [34]. Additionally, the genome contains an acyl-CoA synthetase gene (*acs*, 1_1368) as well as multiple esterase/*αβ*-hydrolase genes (*estA*, 1_436; aes, 1_640, 1_815; *pldB*, 1_203); these enzymes may participate in the activation or cyclization of hydroxy fatty acids. Furthermore, hydroxy fatty acids can spontaneously cyclize to form lactones under acidic fermentation conditions. Previous studies have demonstrated that 4-hydroxydecanoic acid can cyclize into *γ*-decalactone even in dilute acid at room temperature [35]. Therefore, the accumulation of lactones in DZ-fermented butter may result from the combined action of both enzymatic and non-enzymatic mechanisms.

### 3.3. Fatty Acid Metabolism and Genes Associated with Nutritional Remodeling

Previous studies have demonstrated that fermentation with strain DZ can significantly remodel the fatty acid profile of butter: polyunsaturated fatty acids (PUFAs) such as conjugated linoleic acid (CLA), as well as monounsaturated fatty acids (MUFAs) including palmitoleic acid and nervonic acid, are markedly increased; the proportion of oleic acid decreases; the content of medium- and short-chain fatty acids (C4–C12) rises; whereas the proportion of health-risk-associated fatty acids such as palmitic acid declines—thereby facilitating the transformation of butter into a “functional lipid matrix.” To elucidate the molecular basis of these nutritional remodeling phenotypes, this study conducted a systematic analysis of fatty acid metabolism-related genes in the DZ genome.

#### 3.3.1. Genes Involved in Unsaturated Fatty Acid Metabolism

The levels of PUFAs and MUFAs in DZ-fermented butter were both significantly increased. Specifically, CLA increased from 0.55 ± 0.00% to 0.77 ± 0.02% (a 40% increase), *α*-linolenic acid increased from 0.85 ± 0.02% to 1.03 ± 0.02% (a 21% increase), and linoleic acid increased from 2.80 ± 0.08% to 3.29 ± 0.06% (a 17% increase); palmitoleic acid increased by 26%, myristoleic acid by 31%, and nervonic acid by 25%; the remaining various PUFAs and MUFAs also showed increases ranging from 15% to 42%. Meanwhile, the proportion of oleic acid (C18:1) decreased from 23.56 ± 0.14% to 20.42 ± 0.47%.

The oleate hydratase gene (*ohyA*, 1_1392) was identified in the DZ genome; this enzyme converts oleic acid into 10-hydroxystearic acid [36], which is consistent with the decreased proportion of oleic acid. However, oleate hydratase is not directly responsible for CLA formation from linoleic acid. No linoleate isomerase gene was identified in the DZ genome. The increase in CLA may result from other oxidative or stress-related enzymes and non-canonical pathways. The exact enzymatic mechanism requires further experimental validation. The cyclopropane fatty acid synthase gene (*cfa*, 1_682) is involved in the cyclopropanation modification of unsaturated double bonds; this process protects the double bonds of unsaturated fatty acids from adverse reactions such as oxidation, thereby influencing the stability and composition of unsaturated fatty acids [37].

The DZ genome also harbors multiple lipolytic enzyme genes, including GDSL-family lipases (1_319) and *α*/*β*-hydrolases (1_56); these enzymes may participate in the hydrolysis of triglycerides in milk fat, releasing bound unsaturated fatty acids [38,39], thereby providing a precursor source for the overall increase in free unsaturated fatty acids.

Unsaturated fatty acids are, susceptible to oxidative degradation. The DZ genome encodes a complete antioxidant defense system: superoxide dismutase (*sodA*, 1_276) scavenges superoxide anion radicals; alkyl hydroperoxide reductase (*ahpC*, 1_1047; *ahpF*, 1_1046) eliminates lipid peroxides; and glutathione peroxidase (*gpx*, 1_931) reduces hydrogen peroxide and organic peroxides [40]. Compared to non-fermented butter, the continuous action of this system effectively inhibits the oxidative degradation of unsaturated fatty acids, resulting in a significantly higher retention of various PUFAs and MUFAs in DZ-fermented butter than in the non-fermented control.

#### 3.3.2. Regulation of Health-Risk-Associated Fatty Acids

The proportion of palmitic acid (C16:0) decreased from 28.18 ± 0.14% to 24.94 ± 0.48% (a 12% reduction). Palmitic acid is a saturated fatty acid associated with cardiovascular disease risk [41]; its decreased proportion may be linked to changes in the substrate preference of the fatty acid synthase system. Specifically, *β*-ketoacyl-ACP synthase II (*fabF*, 1_548) may facilitate the conversion of palmitic acid into longer-chain fatty acids (e.g., C18:0) by catalyzing the chain elongation reaction of C16:0-ACP, thereby reducing its accumulation level [42]. Additionally, the total amount of trans fatty acids showed a declining trend; the combined levels of elaidic acid and trans-vaccenic acid decreased by approximately 13%, further enhancing the nutritional quality of the butter.

#### 3.3.3. Accumulation of Medium- and Short-Chain Saturated Fatty Acids

Medium- and short-chain fatty acids (C4-C12) play an important role in the functional nutritional profile of butter. Butyric acid (C4:0) exerts anti-inflammatory effects by modulating G protein-coupled receptors and inhibiting histone deacetylases, thereby supporting intestinal health; medium-chain fatty acids such as octanoic acid (C8:0), decanoic acid (C10:0), and lauric acid (C12:0) demonstrate broad-spectrum antibacterial and antiviral activity by disrupting the cell membrane structure of pathogenic microorganisms [43]. In DZ-fermented butter, the concentrations of butyric acid, hexanoic acid, octanoic acid, decanoic acid, and lauric acid increased by 56%, 47%, 36%, 30%, and 22%, respectively. The DZ genome encodes a complete fatty acid synthase system (FAS), including key genes such as *fabH*, *fabD*, *fabG*, *fabZ*, and *fabI*, providing the enzymatic basis for the synthesis of medium- and short-chain fatty acids [44].

### 3.4. Probiotic-Related Genes

The GO functional annotation results (Figure 5) indicate that, in addition to a significant enrichment of genes involved in basic functional categories such as metabolic processes and catalytic activity, the DZ genome also contains genes associated with stress response, transmembrane transport, and localization; these functional categories may collectively provide a molecular basis for its gastrointestinal tolerance characteristics [45].

#### 3.4.1. Acid Resistance-Related Genes

The highly acidic gastric environment (pH 2.5–3.5) represents the primary challenge for probiotics in crossing the gastrointestinal barrier. After being exposed to pH 3.0 for 3 h, strain DZ demonstrated a survival rate of 94.75 ± 1.42%, exhibiting exceptional acid resistance.

Multiple key genes associated with acid tolerance were identified in the DZ genome. Molecular chaperone system genes (*groES*, 1_257; *groEL*, 1_258; *dnaK*, 1_453; *clpC*, 1_381; *clpB*, 1_981; *clpE*, 1_1672) assist in the refolding of denatured proteins under acid stress, protecting cells from damage [46]. The Na^+^/H^+^ antiporter gene (*nhaP*, 1_244, 1_958, 1_985) is one of the core systems employed by bacteria to maintain intracellular pH homeostasis; it resists acidic environments by expelling excess intracellular H^+^ ions [47]. Cation-transporting P-type ATPase genes (*mgtA*, 1_27; *ctpE*, 1_102; *ATP2C*, 1_832, 1_925, 1_1028, 1_2103) play important roles in the response to acid stress by maintaining intracellular cation homeostasis [48]. Detailed information on this gene group, including gene name, locus_tag, replicon ID, function, and associated phenotype, is provided in Supplementary Table S1. The glutamate decarboxylase gene (*gadB*, 1_1622) catalyzes the conversion of glutamate into *γ*-aminobutyric acid (GABA), consuming intracellular H^+^ ions and representing one of the classical acid-tolerance mechanisms in lactic acid bacteria; the synergistic glutamate/GABA antiporter gene (*gadC*, 1_765, 1_1623) imports extracellular glutamate into the cell while exporting intracellularly synthesized GABA out of the cell, thereby ensuring the continuous progression of this metabolic reaction [49].

#### 3.4.2. Bile Salt Resistance-Related Genes

The bile salt concentration in the human small intestine ranges approximately from 0.1% to 0.3%, which can cause damage to the cell membranes of probiotics. After being treated with 0.3% bile salts for 4 h, strain DZ exhibited a survival rate of 40.43 ± 1.75%, demonstrating good bile salt tolerance.

A bile salt hydrolase gene (*cbh*, 1_37) was identified in the DZ genome; this enzyme hydrolyzes conjugated bile salts to generate free bile acids and glycine, thereby reducing the toxic effects of bile salts on the bacterial cell membrane—a key mechanism underlying bacterial bile salt tolerance [50]. Additionally, MATE family efflux pump genes (*mepA*, 1_79, 1_142) and MFS family efflux pump genes (*yebQ*, 1_243; *lmrS*, 1_744) facilitate the efflux of various toxic compounds, including bile salts, providing multiple protective mechanisms for strain DZ against bile salt stress [51].

#### 3.4.3. Antimicrobial Activity-Related Genes

Strain DZ exhibits weak antimicrobial activity against six foodborne pathogens, namely *Staphylococcus aureus*, *Listeria monocytogenes*, Salmonella paratyphi B, *Salmonella typhimurium*, *Shigella dysenteriae*, and *Cronobacter sakazakii*, with inhibition zone diameters ranging from 9.86 ± 0.23 mm to 12.67 ± 0.47 mm. A bacteriocin structural gene (1_1610) was identified in the DZ genome; this gene encodes a bacteriocin precursor peptide and represents a core component of bacteriocin synthesis. The genome also contains ABC transport system genes (1_399, 1_400, 1_933), which may be involved in the extracellular secretion of mature bacteriocins [52]; as well as the enterocin A immunity protein gene (1_524, 1_684), which is primarily responsible for the strain’s self-immunity protection [53].

#### 3.4.4. Genes Related to Intestinal Colonization and Adhesion

The ability of probiotics to colonize the gut is a prerequisite for their beneficial effects. Multiple genes associated with intestinal adhesion and colonization were identified in the DZ genome, suggesting its potential for intestinal colonization.

Adhesion system: The Cna B-type domain protein encoded by the collagen adhesion genes (1_221, 1_1609, 1_1616) mediates the adhesion of strain DZ to the host extracellular matrix collagen [54]. The protein encoded by the fibronectin/fibrinogen-binding protein gene (1_701) binds fibronectin and fibrinogen, facilitating adhesion to host cells and the extracellular matrix [55]. The surface proteins encoded by the LPXTG motif cell wall anchoring protein genes (1_1020, 1_1479, 1_1615, 1_1648) are covalently anchored to the cell wall under the catalysis of sortase A (srtA, 1_718, 1_1422) and serve as crucial molecules mediating bacterial adhesion to host cells [56]. The MucBP domain protein genes (1_1108, 1_1600, 1_1607, 1_1734) encode mucin-binding proteins that specifically bind to mucin—the primary component of the intestinal mucus layer—thereby promoting the colonization of strain DZ on the intestinal surface [57].

Biofilm formation system: The polysaccharide intercellular adhesin synthesis gene (*icaA*, 1_955, 1_1639) participates in biofilm formation [58] and is a key enzyme essential for bacteria to form multicellular aggregates on both biological and abiotic surfaces. The EAL domain protein gene (1_956, 1_1637, 1_1638) encodes a c-di-GMP phosphodiesterase that regulates biofilm formation and disassembly through the degradation of the second messenger c-di-GMP [59]. The quorum sensing signal molecule synthesis gene (*luxS*, 1_1873) synthesizes the AI-2 quorum sensing molecule and regulates the expression of genes involved in biofilm formation and gut colonization [60].

Surface property modification system: The D-alanine–lipoteichoic acid modification system (dlt operon, 1_1024–1_1027) comprises *dltA* (D-alanine activation), *dltB* (membrane protein), *dltC* (D-alanine carrier protein), and *dltD* (D-alanine transfer protein). This gene cluster facilitates the incorporation of D-alanine into lipoteichoic acid, thereby reducing the negative surface charge of the cell, increasing its surface hydrophobicity and self-aggregation capacity, and thereby enhancing initial bacterial adhesion to host cells. Additionally, this modification enhances the resistance of strain DZ to bile salts and cationic antimicrobial peptides, making it an important contributor to the colonization capability of probiotics [61].

#### 3.4.5. Antioxidant-Related Genes

In the gastrointestinal environment, probiotics not only face stress from acid and bile salts but also must combat oxidative damage induced by reactive oxygen species (ROS) generated by cellular metabolism and environmental factors. If ROS are not promptly eliminated, they can damage cellular proteins, DNA, and cell membranes [62]. The DZ genome encodes a complete antioxidant defense system, suggesting its potential antioxidant capacity.

The superoxide dismutase gene (*sodA*, 1_276) scavenges superoxide anion radicals and serves as the first line of defense against oxidative stress. The alkyl hydroperoxide reductase gene (*ahpC*, 1_1047; *ahpF*, 1_1046) and the organic hydroperoxide reductase gene (*osmC*, 1_26, 1_866, 1_975) scavenge hydrogen peroxide and organic peroxides. The glutathione peroxidase gene (*gpx*, 1_931) reduces hydrogen peroxide, thereby protecting cell membrane lipids from oxidative damage [63].

The thioredoxin reductase gene (*trxB*, 1_622, 1_760) and the thioredoxin gene (*trxA*, 1_253, 1_625) help maintain the intracellular redox balance [64]. The methionine sulfoxide reductase gene (msrA, 1_525, 1_1804; *msrB*, 1_1090) can repair proteins damaged by oxidative stress and restore their function [65].

These antioxidant genes provide strain DZ with a safeguard against oxidative damage in the gastrointestinal environment, while also helping to protect the polyunsaturated fatty acids it produces from oxidative degradation.

### 3.5. Biosafety Assessment

The NR database annotation results (Figure 6) show that 83.06% of the genes in the DZ genome exhibit the highest similarity to those of *Lactococcus garvieae*, confirming the species identification of this strain.

#### 3.5.1. Hemolytic Activity Analysis

The metabolism of lactic acid bacteria may generate hemolytic toxins that induce erythrocyte lysis; therefore, hemolytic assay serves as an important screening indicator for microbial safety in food-grade products [66]. Strain DZ showed no hemolysis on Columbia blood agar plates, indicating γ-hemolysis (non-hemolytic). The positive control, Pseudomonas aeruginosa, exhibited typical β-hemolysis (a clear hemolytic zone), whereas the negative control showed no hemolysis. Notably, although the hemolysin III gene (*hlyIII*, 1_317) was identified in the DZ genome, this gene was annotated in the database solely as a “predicted membrane protein” (COG1272) rather than as a typical hemolytic toxin gene. Its encoded product was annotated as an integral membrane protein containing a hemolysin-III related domain, but no known toxin catalytic domain was identified. Therefore, the genomic annotation does not support a functional hemolytic role for HlyIII. The non-hemolytic phenotype of strain DZ is supported primarily by the experimental blood agar result, indicating that strain DZ does not possess hemolytic virulence.

#### 3.5.2. Antibiotic Resistance Genes and Virulence Factor Analysis

The disk diffusion method was employed to determine the susceptibility of strain DZ to 10 different antibiotics. The results showed that strain DZ was susceptible to ampicillin, tetracycline, chloramphenicol, erythromycin, clindamycin, gentamicin, ciprofloxacin, and vancomycin; however, it exhibited resistance to streptomycin and rifampicin only.

The CARD database annotation results (Figure 7) indicate that no transferable acquired resistance genes associated with the aforementioned sensitive antibiotics (e.g., tetM, ermB, cat, etc.) were detected in the DZ genome, including both the chromosome and the three plasmids (pDZ1–pDZ3), which is consistent with its sensitive phenotypic profile. VFDB annotation of the DZ genome, also covering the chromosome and the three plasmids, identified only four genes with homology to virulence-associated factors: rmlB (capsule biosynthesis), tig/ropA (trigger factor), SSU98_1513 (phosphopyruvate hydratase), and plr/gapA (glyceraldehyde-3-phosphate dehydrogenase). Importantly, none of these genes encodes a typical toxin. They are auxiliary metabolic or housekeeping genes that are widely distributed in non-pathogenic lactic acid bacteria. For example, rmlB is involved in capsule polysaccharide synthesis, tig/ropA is a molecular chaperone, and SSU98_1513 and plr/gapA are glycolytic enzymes that can bind host proteins in some pathogenic species but are not toxins themselves. No hemolysin or enterotoxin gene was detected, consistent with the γ-hemolytic phenotype. Compared with pathogenic L. garvieae lineages, strain DZ lacks the key virulence modules required for full pathogenicity. However, these genomic findings do not rule out the possibility of environment-induced virulence expression or horizontal gene transfer, and further strain-level safety evaluation is required.

It is noteworthy that strain DZ is sensitive to vancomycin. Some Lactococcusand *Weissella* strains may carry van resistance gene clusters, which modify the cell wall precursor terminus from D-Ala-D-Ala to D-Ala-D-Lac, thereby conferring vancomycin resistance. No complete van resistance gene cluster was identified in the DZ genome; only a few van family homologs were detected. The D-alanine–D-alanine ligase gene (*ddl*, 1_205) synthesizes the normal D-Ala-D-Ala terminus, making this strain sensitive to vancomycin. This represents an important safety characteristic that distinguishes this strain from most resistant Lactococcus and *Weissella* species.

The resistance of strain DZ to streptomycin and rifampicin may be associated with intrinsic chromosomal target genes. The ribosomal protein S12 gene (rpsL, 1_2116), the 16S rRNA methyltransferase gene (rsmG, 1_673), and the RNA polymerase *β* subunit gene (rpoB, 1_422) were identified in the DZ genome. These genes are known targets of streptomycin and rifampicin, respectively, and mutations in these genes have been reported to confer resistance in other bacteria [67,68]. However, we did not perform sequence alignment or mutation analysis in this study; therefore, the exact resistance mechanism in strain DZ remains to be experimentally confirmed. Because these genes are located on the chromosome and no acquired resistance genes were detected, the observed resistance is likely intrinsic rather than acquired, and the risk of horizontal transfer is expected to be low.

## 4. Conclusions

This study completed the whole-genome sequencing and functional annotation of *Lactococcus garvieae* DZ and, by integrating prior phenotypic data, systematically elucidated at the genetic level the mechanisms underlying this strain’s superior fermentation performance, flavor formation, nutritional remodeling, and probiotic properties.

The DZ genome consists of one circular chromosome (2,146,103 bp, GC content 38.81%) and three plasmids (15.3–74.1 kb), encoding a total of 2123 genes. No typical transposable elements or simple repetitive sequences were identified in the genome; only a small number of long fragment repeats were present, indicating that this strain possesses high genetic stability and is well-suited as an industrial fermentation starter strain.

In terms of flavor formation, the genome fully encodes the complete metabolic pathway from lactose degradation to pyruvate metabolism, as well as key enzyme systems involved in diacetyl/acetoin synthesis, the accumulation of short- and medium-chain fatty acids (C4–C10), and ester synthesis. The synergistic expression of these genes explains the rapid accumulation of characteristic flavor compounds—such as 2,3-butanedione (6188 μg·kg^−1^) and 2,3-pentanedione (4660 μg·kg^−1^)—as well as the substantial increase in various short- and medium-chain fatty acids (hexanoic acid and octanoic acid levels increased by 3.6-fold) in DZ-fermented butter, thereby providing clear genetic targets for flavor profile modulation in high-quality fermented butter.

In terms of nutritional remodeling, the genetic configuration of the fatty acid synthase system (FAS) and the medium-chain acyl-ACP thioesterase (*tesA*) provides enzymatic support for the enrichment of medium- and short-chain fatty acids. The discovery of oleate hydratase (*ohyA*) explains the decrease in oleic acid proportion (23.56%→20.42%), while the increase in conjugated linoleic acid (40%) may involve other oxidative or stress-related enzymes; the exact mechanism requires further validation. The complete encoding of antioxidant-related genes—such as superoxide dismutase (*sodA*) and alkyl hydroperoxide reductase (*ahpC*)—provides a molecular basis for the oxidative protection of polyunsaturated fatty acids during the fermentation process. Together, these genes constitute the enzymatic network through which strain DZ remodels conventional butter into a “functional lipid matrix.”

Regarding probiotic properties and safety, genes identified in the genome—such as the Na^+^/H^+^ antiporter (*nhaP*), bile salt hydrolase (*cbh*), and molecular chaperone system (*groES*/*groEL*)—are highly consistent with the strain’s excellent gastrointestinal tolerance (94.75% survival at pH 3.0 and 40.43% survival in the presence of 0.3% bile salts); the discovery of adhesion-related genes (MucBP, LPXTG motif protein, collagen adhesins) and biofilm formation-associated genes (*icaA*, *luxS*) suggests that this strain possesses potential for intestinal colonization. In terms of safety, VFDB annotation identified only four genes with homology to virulence-associated factors, none of which encodes a typical toxin, and no typical functional hemolysin gene was identified, consistent with the *γ*-hemolytic phenotype. The resistance to streptomycin and rifampicin may be associated with intrinsic chromosomal target genes (*rpsL*, *rsmG*, *rpoB*); the exact resistance mechanism requires further experimental validation. However, similar to other L. garvieae strains from fermented foods, the possibility of environment-induced virulence expression and horizontal gene transfer cannot be excluded.

In conclusion, strain DZ shows excellent fermentation performance and potential for nutritional remodeling. Using the databases and thresholds employed in this study, no known acquired resistance determinants or established virulence-associated genes were detected. However, *L. garvieae* has not been granted QPS status by EFSA, and these findings do not by themselves establish that the strain is food-grade or probiotic. In addition, the possibility of horizontal gene transfer cannot be excluded, and further studies are needed to assess the potential for transfer of genetic elements under different environmental conditions. Further strain-level safety evaluation is required before industrial application. This study not only provides a theoretical foundation for the industrial application of strain DZ but also identifies clear genetic modification targets for optimizing diacetyl synthesis, enhancing conjugated linoleic acid accumulation, and unlocking its probiotic potential through metabolic engineering. Strain DZ therefore shows great potential for development as a high-quality fermented butter starter culture and functional probiotic formulation with independent intellectual property rights.

## Figures and Tables

**Figure 1:**
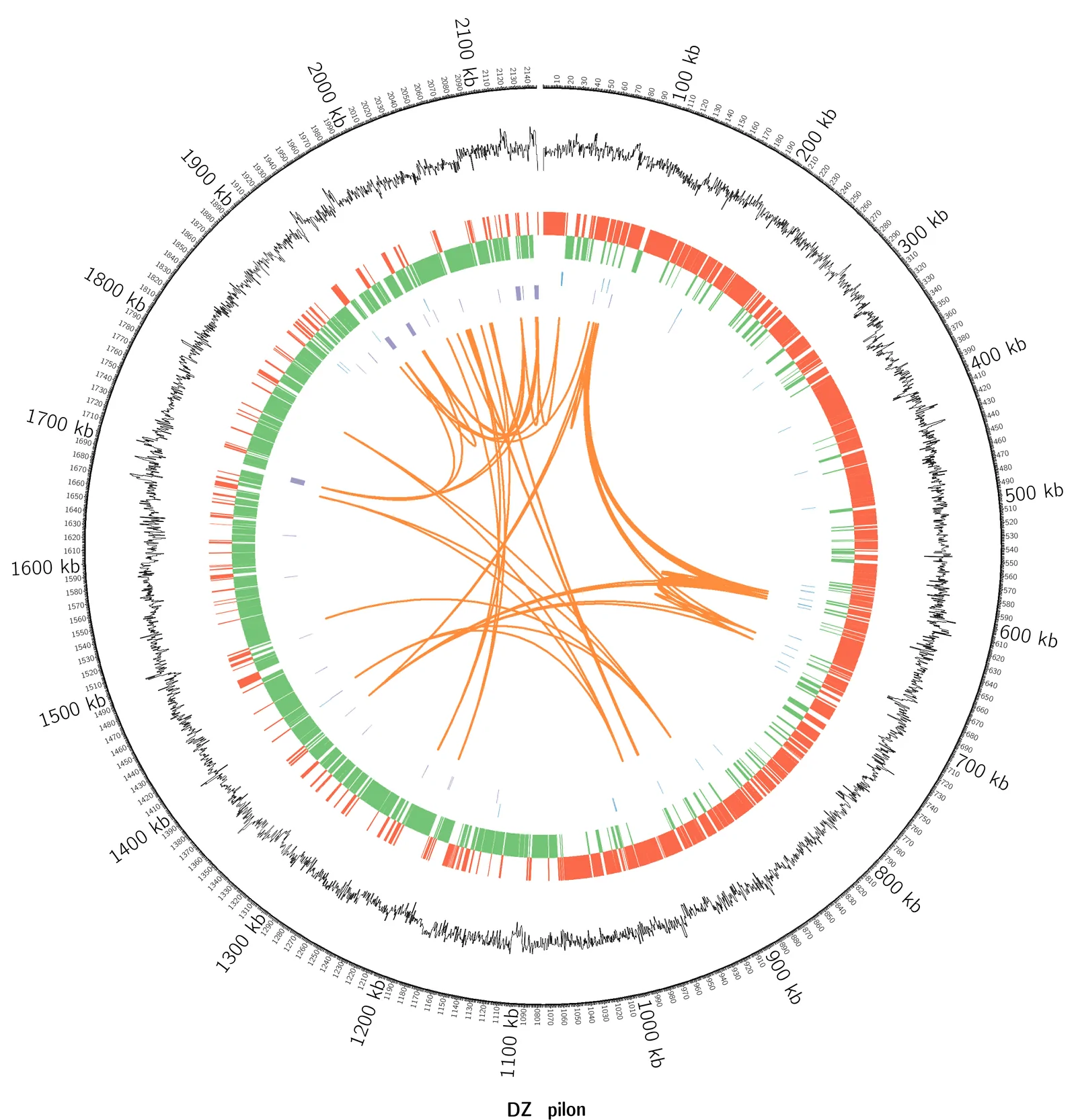
Circular genome map of *Lactococcus garvieae* DZ. Note: The circle diagram from outside to inside shows: 1st ring, genomic position information; 2nd ring, GC content (black curve); 3rd ring, coding genes on the positive strand (red); 4th ring, coding genes on the negative strand (green); 5th ring, ncRNA information on the positive strand (blue); 6th ring, ncRNA information on the negative strand (purple); 7th ring, long fragment repeat sequences within the genome (marked in orange).

**Figure 2:**
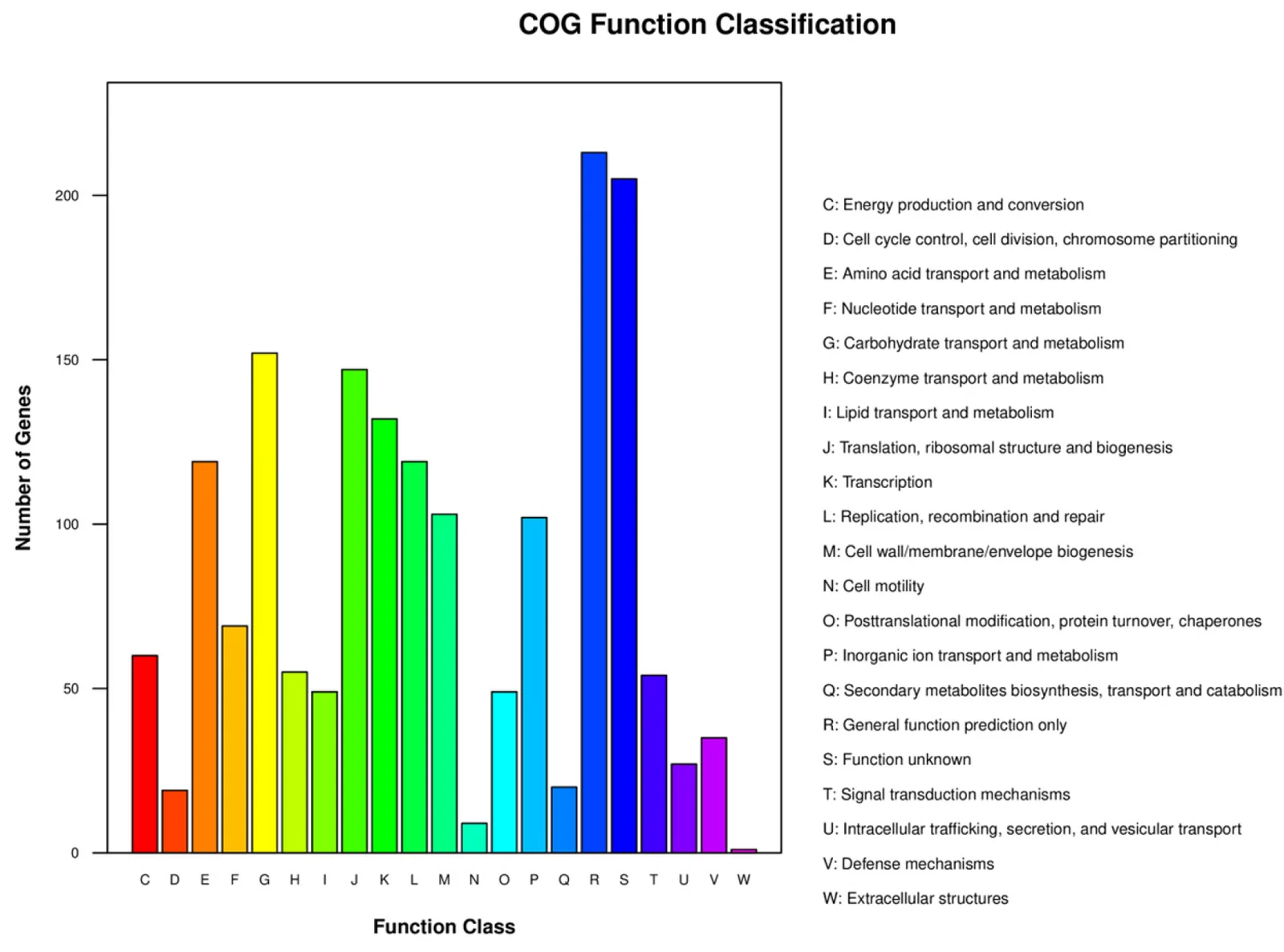
COG functional classification of the *Lactococcus garvieae* DZ genome. Note: The horizontal axis represents COG functional categories; the vertical axis represents the number of genes annotated in each category; detailed explanations of each category are provided on the right side of the figure.

**Figure 3:**
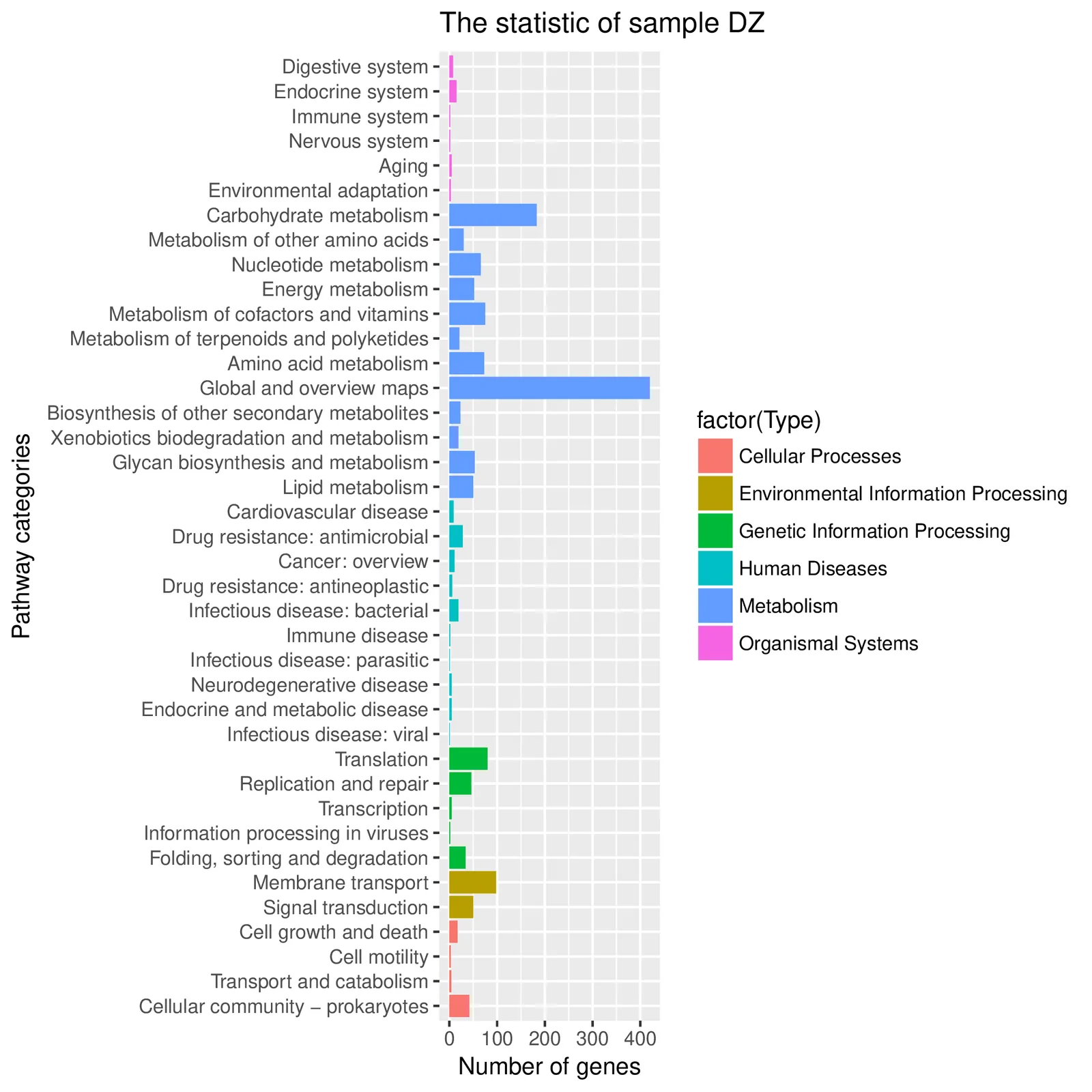
KEGG metabolic pathway classification statistics of the *Lactococcus garvieae* DZ genome. Note: The vertical axis represents the secondary classification of biological pathways; the horizontal axis indicates the number of genes; different colors distinguish the primary classifications of the biological pathways.

**Figure 4:**
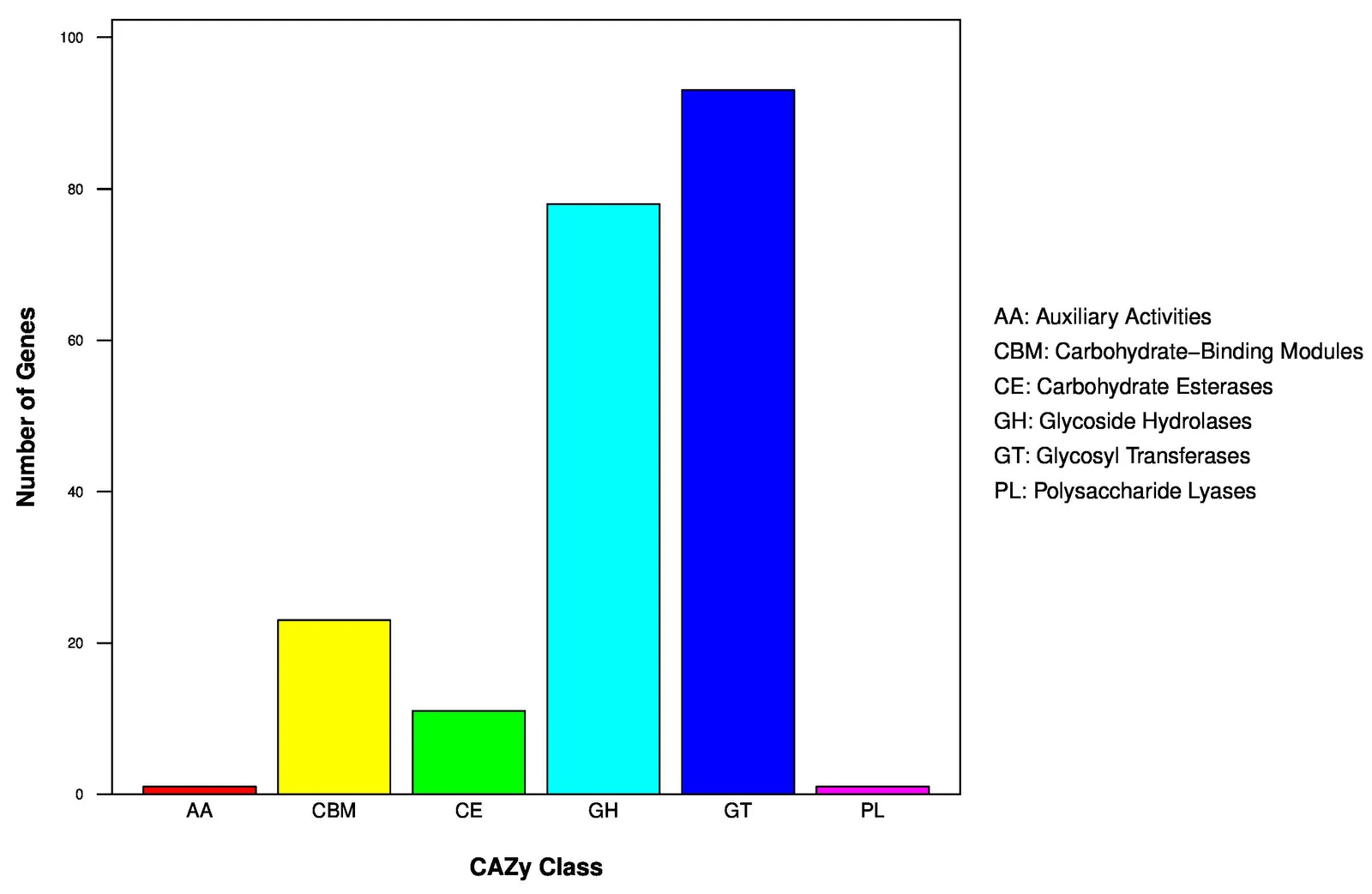
CAZy carbohydrate-active enzyme classification of *Lactococcus garvieae.* Note: The horizontal axis represents the CAZy Class classification; the vertical axis represents the number of genes.

**Figure 5:**
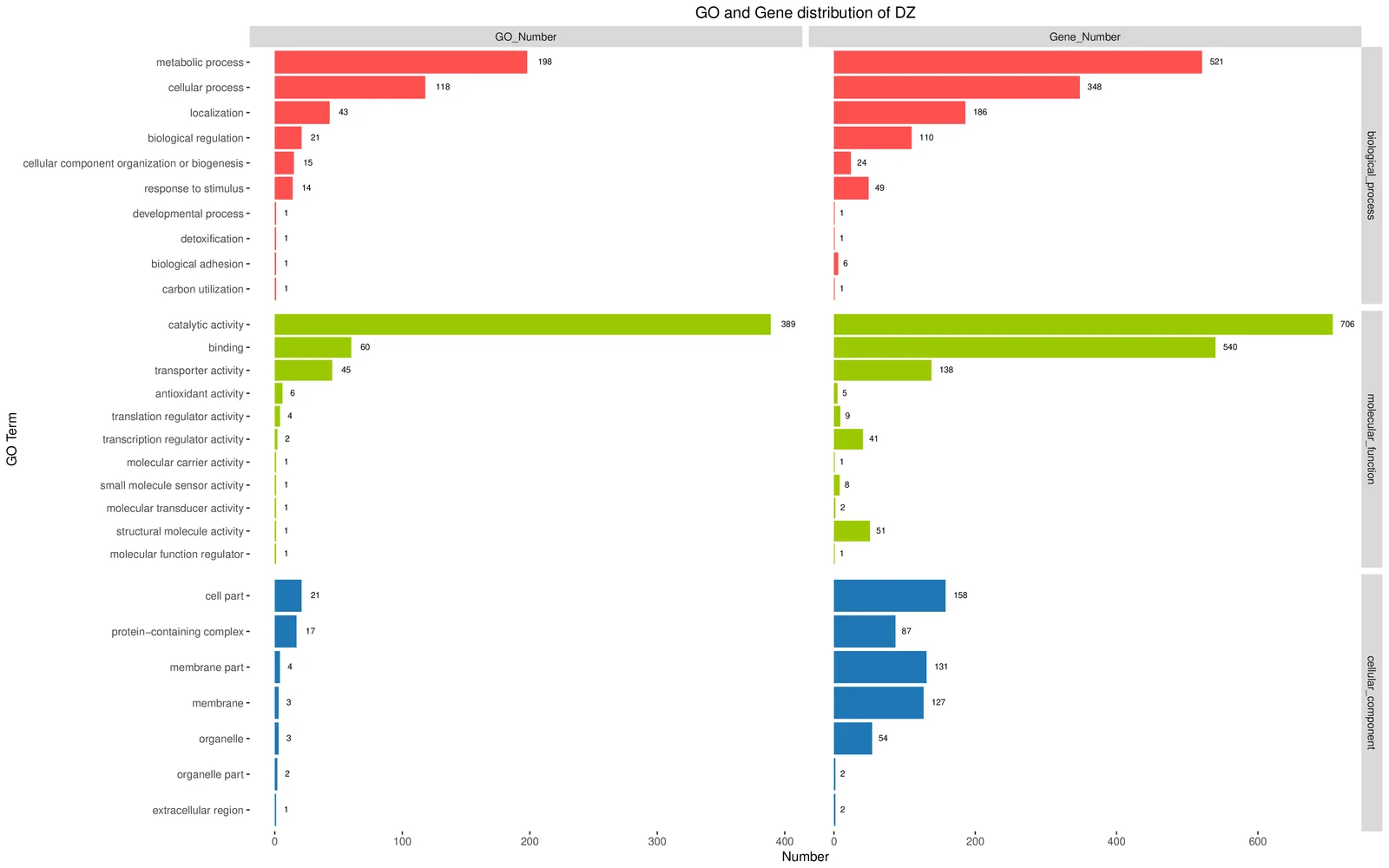
GO functional annotation distribution of the *Lactococcus garvieae* DZ genome. Note: The vertical axis represents GO terms; the horizontal axis represents the number of genes. Different colors correspond to the three major ontologies: Biological Process (BP), Cellular Component (CC), and Molecular Function (MF).

**Figure 6:**
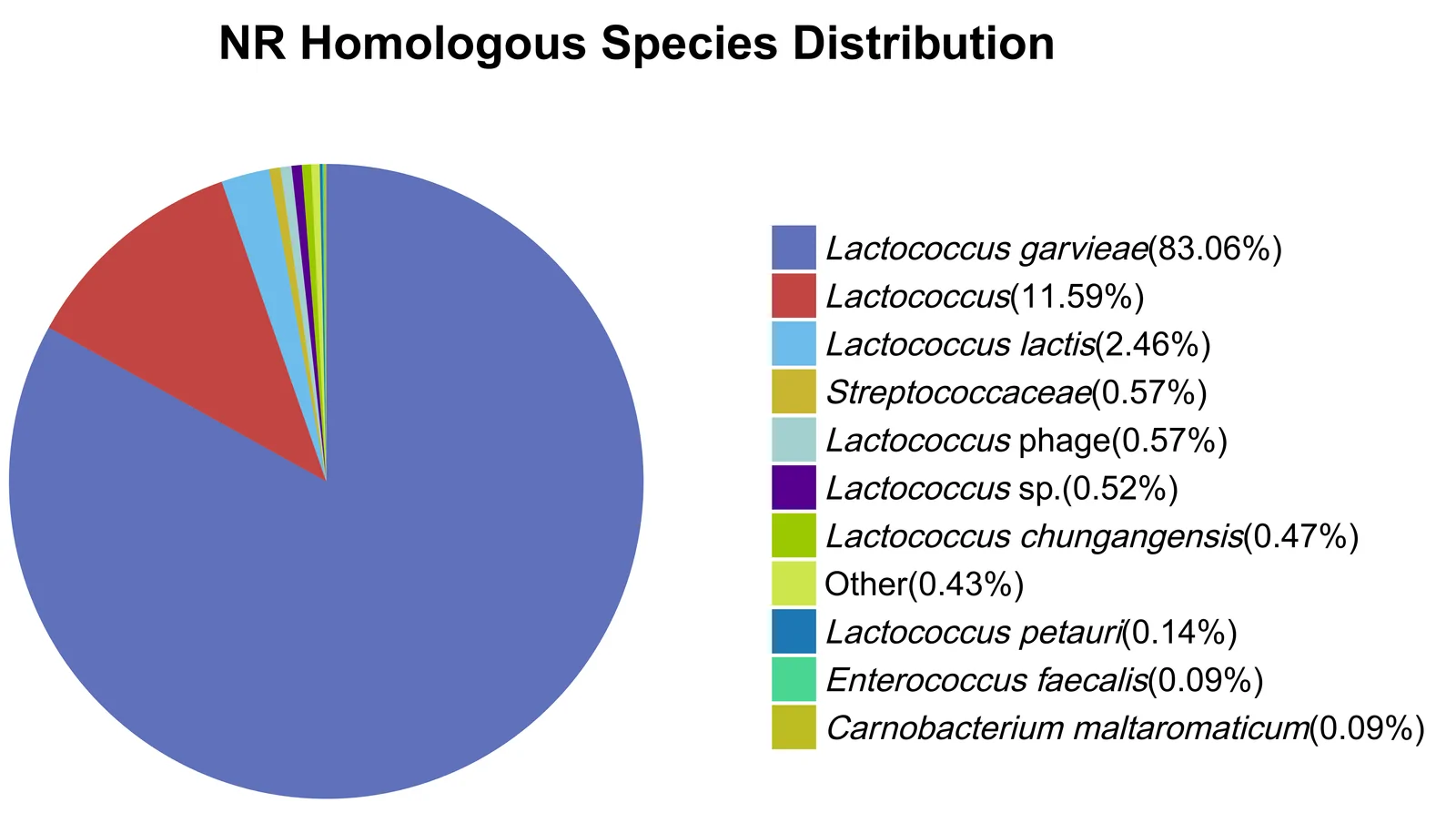
NR species distribution of the *Lactococcus garvieae* DZ genome. Note: The pie chart illustrates the distribution proportions of different species; different colors represent different species. Percentages may not sum to 100% due to rounding.

**Figure 7:**
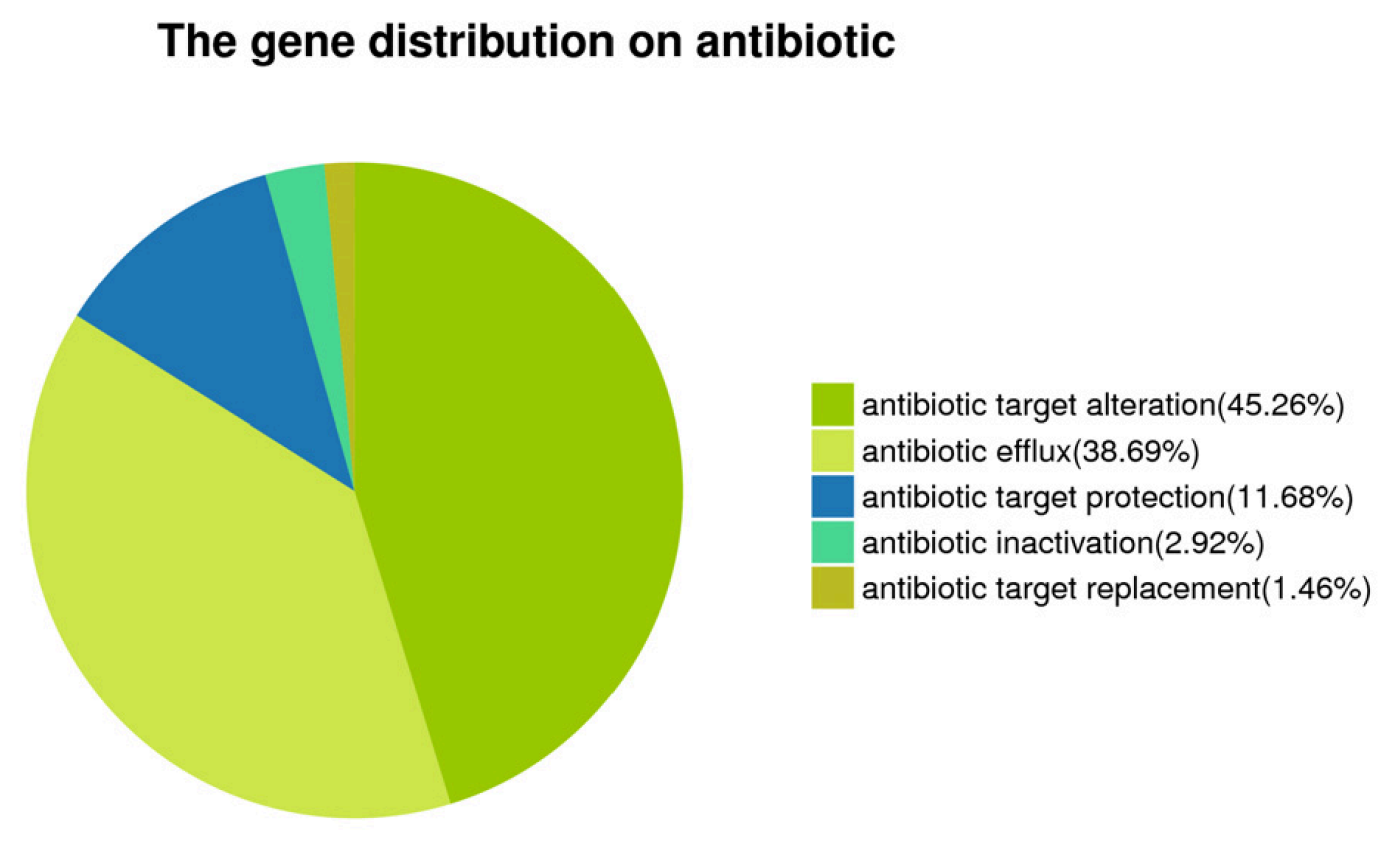
CARD resistance gene functional classification of the *Lactococcus garvieae* DZ genome. Note: Different colors in the pie chart represent different ARO categories; the size of each sector indicates the relative proportion of genes within that category. Percentages may not sum to 100% due to rounding.

## Data Availability

The genome sequence data of *Lactococcus garvieae* DZ have been deposited in the Genome Warehouse (GWH) of the China National Center for Bioinformation (CNCB) under the accession number GWHJNDM00000000.1. The BioSample accession number is SAMC8067568. Both the GWH accession number and the BioSample number have been verified to be live and accessible. The assembled genome is available at https://ngdc.cncb.ac.cn/gwh (accessed on 20 September 2026) under the accession GWHJNDM00000000.1.

## Supplementary Materials

The following supporting information can be downloaded at: https://www.mdpi.com/article/10.3390/microorganisms14102163/s1, Table S1: Annotation of the *mgtA*/*ctpE*/*ATP2C* gene group in *Lactococcus garvieae* DZ.
