# Supplementary material for: Whole-Genome Sequencing Elucidates the Superior Fermentation Performance, Flavor Formation, and Nutritional Remodeling Mechanisms of Lactococcus garvieae DZ for Fermented Butter Production
Source: Microorganisms. 2026 Sep 25;14(10):2163. doi: 10.3390/microorganisms14102163 (PMC13707513; doi:10.3390/microorganisms14102163)
Supplement: Supplementary file 1 [file microorganisms-14-02163-s001.zip › microorganisms-4573221-supplementary.pdf]

**Supplementary Table S1.** Annotation of the *mgtA/ctpE/ATP2C* gene group *in Lactococcus garvieae* DZ.

| Gene name | Locus_tag | Replicon ID | Function | Associated phenotype |
|---|---|---|---|---|
| *mgtA* | 1_27 | Chromosome | Magnesium-translocating P-type ATPase / P-type $Mg^{2+}$ transporter | Acid stress response (maintenance of intracellular cation homeostasis) |
| *ctpE* | 1_102 | Chromosome | Cation-transporting P-type ATPase E | Acid stress response (maintenance of intracellular cation homeostasis) |
| *ATP2C* | 1_832 | Chromosome | P-type $Ca^{2+}$ transporter type 2C | Acid stress response (maintenance of intracellular cation homeostasis) |
| *ATP2C* | 1_925 | Chromosome | P-type $Ca^{2+}$ transporter type 2C | Acid stress response (maintenance of intracellular cation homeostasis) |
| *ATP2C* | 1_1028 | Chromosome | P-type $Ca^{2+}$ transporter type 2C | Acid stress response (maintenance of intracellular cation homeostasis) |
| *ATP2C* | 1_2103 | Chromosome | P-type $Ca^{2+}$ transporter type 2C | Acid stress response (maintenance of intracellular cation homeostasis) |

Supplementary Table S1. Annotation of the *mgtA/ctpE/ATP2C* gene group in *Lactococcus garvieae* DZ. All genes in this group encode cation-transporting P-type ATPases located on the chromosome and are associated with acid stress response through the maintenance of intracellular cation homeostasis.
